# Statistical techniques to construct assays for identifying likely responders to a treatment under evaluation from cell line genomic data

**DOI:** 10.1186/1471-2407-10-586

**Published:** 2010-10-27

**Authors:** Erich P Huang, Jane Fridlyand, Nicholas Lewin-Koh, Peng Yue, Xiaoyan Shi, David Dornan, Bart Burington

**Affiliations:** 1 Biometric Research Branch - Department of Cancer Treatment and Diagnosis, National Cancer Institute, National Institutes of Health. Rockville, MD 20852, USA; 2 Genentech, Inc. South San Francisco, CA 94080, USA

## Abstract

**Background:**

Developing the right drugs for the right patients has become a mantra of drug development. In practice, it is very difficult to identify subsets of patients who will respond to a drug under evaluation. Most of the time, no single diagnostic will be available, and more complex decision rules will be required to define a sensitive population, using, for instance, mRNA expression, protein expression or DNA copy number. Moreover, diagnostic development will often begin with in-vitro cell-line data and a high-dimensional exploratory platform, only later to be transferred to a diagnostic assay for use with patient samples. In this manuscript, we present a novel approach to developing robust genomic predictors that are not only capable of generalizing from in-vitro to patient, but are also amenable to clinically validated assays such as qRT-PCR.

**Methods:**

Using our approach, we constructed a predictor of sensitivity to dacetuzumab, an investigational drug for CD40-expressing malignancies such as lymphoma using genomic measurements of cell lines treated with dacetuzumab. Additionally, we evaluated several state-of-the-art prediction methods by independently pairing the feature selection and classification components of the predictor. In this way, we constructed several predictors that we validated on an independent DLBCL patient dataset. Similar analyses were performed on genomic measurements of breast cancer cell lines and patients to construct a predictor of estrogen receptor (ER) status.

**Results:**

The best dacetuzumab sensitivity predictors involved ten or fewer genes and accurately classified lymphoma patients by their survival and known prognostic subtypes. The best ER status classifiers involved one or two genes and led to accurate ER status predictions more than 85% of the time. The novel method we proposed performed as well or better than other methods evaluated.

**Conclusions:**

We demonstrated the feasibility of combining feature selection techniques with classification methods to develop assays using cell line genomic measurements that performed well in patient data. In both case studies, we constructed parsimonious models that generalized well from cell lines to patients.

## Background

Targeted therapies and individualized medicine have become buzz-words in drug development [1]. However, in practice it is extremely difficult to identify molecular subpopulations expected to respond to an investigational drug. Trastuzamab, for Her2-positive breast cancer patients [2], and imatinib, for chronic myeloid leukemia (CML) driven by 9/22 translocation also known as Philadelphia chromosome [3], represent rare success stories for personalized treatment. However, the targeted population for these drugs was defined pre-clinically based on overwhelming scientific evidence. Even for the case of trastuzamab, where a single diagnostic marker is known, the most appropriate assay is still unclear, with a combination of two assays defining the current clinical practice.

In most cases, however, a single diagnostic marker is not available, and more complex decision rules will be required to define a sensitive population based upon, for instance, mRNA expression, protein expression or DNA copy number. This was recognized by the FDA Critical Path Initiative [1] which calls for development of new biomarkers, asserting that *new microarray technologies, that can rapidly analyze the expression of thousands of genes, may make it possible to identify sets of biomarkers that are more predictive of clinical risks or benefits than single markers for a given condition*. However, development of assays to identify likely responders to a drug based on gene expression measurements has some inherent difficulties. First, development of such assays often need to be performed on cell lines rather than clinical samples, which in many cases are not available until later in the clinical development cycle. Second, such assays must only involve a small number of genes. High dimensional biomarkers or *signatures*, biomarkers that depend on the expression levels of a large number of genes, are generally not robust to inherent assay variability. Furthermore, classifiers involving small numbers of genes are conducive to PCR-based assays; even though development of the classifier may occur on a microarray, the final diagnostic assay is likely to be PCR-based. Patients routinely undergo a tissue biopsy for diagnosis and the biopsy is subsequently Formalin Fixed and Paraffin Embedded (FFPE). FFPE tissue is renowned for having poor quality RNA due to extensive degradation as a result of paraffin embedding as well as extensive cross-links from formalin fixation which can also affect the quality of RNA extracted. qRT-PCR assays have been successfully implemented in clinical practice from fixed tissue for multi-gene assays, but such PCR-based assays can only feasibly involve 30 or fewer genes.

In addressing the development problems described above, this manuscript deals with two statistical issues inherent to the problem of developing a robust diagnostic assay: *feature selection *and *model fitting*. A great deal of research has gone into statistical techniques for the selection of lower-dimensional subsets of variables for prediction, particularly for cases where the number of classifiers *p *is far greater than the number of samples *N*, a phenomenon that is characteristic of microarray data; many oncologic indications only have a limited number of the cell lines at a time (50 - 100), yet the expressions of tens of thousands of genes are measured for each cell line. One prominent example of such a technique is the Lasso [4], which involves finding coefficients under the standard multivariate linear regression model that maximize the log-likelihood subject to a constraint on the *L*_1_-norm of the coefficients, namely the sum of the absolute values of the coefficients. The effect of the *L*_1_-norm constraint is that many of the coefficients will be set to exactly zero, thus resulting in feature selection; we describe this technique in more detail in the Methods section. Another example is what we call Self-Normalizing Stepwise Selection (SNSS), a forward stepwise procedure that adds variables to a simple signed average according to their strength of association with the outcome.

Our aim is to explore combinations of feature selection methods with various alternate model fitting techniques in constructing assays with high responder versus non-responder classification accuracy. We apply a feature selection method such as Lasso or SNSS to cell line data to find the subset of genes that best predict a cell line's disease subtype or sensitivity to a drug, and then employ alternate classification techniques, such as K-Nearest Neighbors (KNN) [5] or Random Forests [6], to develop classifiers that we will compare in terms of responder versus non-responder classification accuracy.

We apply this approach to two case studies. In the first, we use cell line gene expression and sensitivity to dacetuzumab (SGN-40) [7,8], a drug targeting malignant B-cells, to construct a predictive model of diffuse large B-cell lymphoma (DLBCL) patient sensitivity to the same drug. In the second, we use gene expression data from breast cancer cell lines to develop classifiers of estrogen receptor (ER) status for application in breast cancer patients. The molecular classification of breast cancer is of high importance due to patient subtype-specific prognoses and the subtype-specificity of potential drug targets [9,10]. While the IHC assay for ER status is a widely accepted standard, we used expression-based ER prediction as another case study for comparing the performance of classifiers.

For the rest of the paper, we will assume that the genomic data represents genome-wide mRNA expression measurements, and we will use the terms *biomarker*, *gene signature*, and *classifier *interchangeably. The methods we develop are not specific to any technology or experimental setting.

### The Data and the Two Case Studies

As the first case study, we apply the proposed methodology to predict, based on the cell line data, the sensitivity of the diffuse large B-cell lymphoma (DLBCL) patients to dacetuzumab, a drug for DLBCL developed jointly by Seattle Genetics, Inc. and Genentech, Inc.

Non-Hodgkin's lymphoma (NHL) consists of a diverse group of lymphoid neoplasms that, in the United States, rank sixth in estimated incidence of new cancer cases and ninth and sixth in estimated cancer mortality among men and women, respectively. It is estimated that in 2009, NHL accounted for 3% and 4% of cancer deaths of men and women, respectively, in the United States [11]. Diffuse large B-cell lymphoma (DLBCL), the most common histologic subtype of Non-Hodgkin's lymphoma (NHL), accounts for approximately 30 percent of NHL cases. DLBCL arises from a mature B-cell, the majority of which express a CD20+ cell-surface protein. Several genetic abnormalities have been identified in subsets of DLBCL. The most frequently dysregulated genes include BCL-6, BCL-2, and cMYC [12].

Dacetuzumab targets the B-cell CD40 pathway, which acts as a trigger for the transformation of germinal center B-cells into activated B-cells. Pre-clinical evidence suggests that dacetuzumab is most active in DLBCL cell lines with inactive CD40 pathways [8]. In these cell lines, dacetuzumab may stimulate the pathway, initiating cell signaling cascades that interfere with the functioning of the tumor cell. On the other hand, cell lines with activated CD40 pathways may be less sensitive to further signaling by dacetuzumab. Previously, Alizadeh et al divided DLBCL samples into germinal center B-cell like (GCB) and activated B-cell like (ABC) subtypes using gene expression profiling [13]. The GCB and ABC subtypes are strongly correlated with inactive or active CD40 pathways in cell lines. In addition, the GCB subtype has been reported to be associated with better overall and progression-free survival. Hence, there is a strong prior scientific case that groups of patients predicted to respond to dacetuzumab therapy should overlap with the GCB subtype malignancy, and hence the signature for Dacetuzumab sensitivity should be enriched for the patients of the GCB subtype and with better survival.

**Figure 1:**
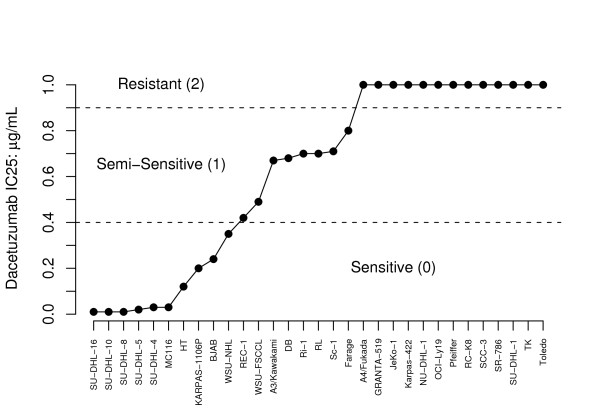
**Plots of sensitivities to dacetuzumab of each of the 31 cell lines used in this case study**. A plot of the dacetuzumab IC25 (in *μ*g/mL) of each of the 31 cell lines used in the dacetuzumab sensitivity case study, taken from results originally reported by Burington et al (Burington, B., Yue, P., Shi, X., et al: CD40 Pathway Activation Status Predicts the Anti-Tumor Activity of CD40 Therapy in Diffuse Large B-Cell Lymphoma, submitted). The IC25 is defined as the dosage required to kill 25% of the cells. A maximum dosage of 1 *μ*g/mL was administered to each of the cell lines. We define a cell line as sensitive if the IC25 is below 0.4 *μ*g/mL, semi-sensitive if the IC25 is between 0.4 and 1 *μ*g/mL, and resistant if the maximum dosage was insufficient to kill 25% of the cells.

The cell line data consist of sensitivities to dacetuzumab and approximately 50 thousand Affy U133Plus2.0 platform probe set measurements of the gene expressions for 31 cell lines. We have three replicates of the probe set measurements for each of the cell lines. Each cell line was treated with up to 1 *μ*g per mL of dacetuzumab; a cell line was labeled as dacetuzumab sensitive if the IC25, the dosage required to kill 25% of the cells, was less than 0.4 *μ*g per mL, as dacetuzumab intermediate or semi-sensitive if the IC25 was greater than 0.4 *μ*g per mL but less than 1 *μ*g per mL, and as dacetuzumab resistant if 1 *μ*g per mL was insufficient to kill 25% of the cells (see Figure 1). Meanwhile, the previously published observational patient data include microarray measurements for 20 thousand probe sets on a U133A platform, DLBCL subtypes (ABC, GCB, or unclassifiable), and survival data (number of months until their follow-up and status, namely dead or alive, at time of follow-up) for 221 patients (GEO Accession ID GSE4475) [14].

In the second case study, we examine the performance of our methodologies in developing classifiers for estrogen receptor (ER) positivity. While the IHC-based assay for ER positivity is well established, we use ER status prediction as a base case for the classifiers.

To construct ER status classifiers, we use cell line genomic data consisting of measurements of approximately 22 thousand probe sets on an Affy U133A platform for 65 cell lines (ArrayExpress Accession ID E-TABM-157) [15]. However, for eighteen of these cell lines, the ER status is missing; we only use data for the remaining 47 cell lines to construct our classifiers. The ER status assigned to each of these cell lines was based on previously published literature using copy number, gene and protein expression data.

We test these classifiers on a data set containing Affy U133A measurements for 118 breast cancer patients (ArrayExpress Accession ID E-TABM-158) [10]. The ER status for each patient is known based on the clinical assay. Both cell lines and tumor breast cancer datasets referenced above are described in the Cancer Cell companion manuscripts [10,15]. These papers present a comprehensive review of the variety of genomic alterations arising in breast tumors and demonstrate the concordance of these alterations between primary tumors and cell lines as well as examine the relevance of the observed changes to clinical phenotypes.

## Methods

**Figure 2:**
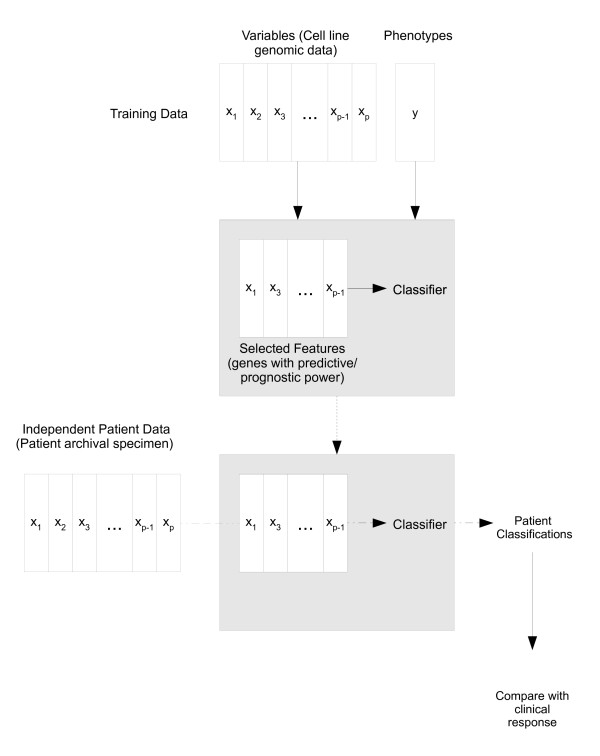
**Schematic of analysis procedure**. We use the cell line data to construct a classifier of dacetuzumab sensitivity or ER status given gene expressions, and we subsequently apply this classifier to patient genomic data to classify these patients according to phenotype.

Figure 2 is a schematic of the overall procedure. Using the cell line data, we perform feature selection followed by model fitting, thus forming classifiers for dacetuzumab sensitivity or ER status. These classifiers are then applied to patient data, allowing assessment of the performance of each method.

### Feature Selection

In our feature selection procedures, we search for a small subset of genes with predictive or prognostic power out of the genome-wide candidate pool. For this, we consider three techniques:

**• Lasso **[4]. Here, we adopt a general linear model of phenotype with respect to gene expressions:

(1) $$g\left(Y\right)=\sum_{j=1}^{p}\beta_{j}X_{j}$$

*Y *denotes a numeric coding of the phenotype and *X_j _*the measurement for probe set *j*. For the dacetuzumab sensitivity example, *Y *= 0 denotes sensitivity, *Y *= 1 semi-sensitivity, and *Y *= 2 resistance, whereas for the ER status example, *Y *= 1 denotes ER positivity and *Y *= 0 denotes ER negativity. *g*, meanwhile, is a *link function*, whose form depends on the characteristics of *Y *. For ordered or quantitative phenotypes such as dacetuzumab sensitivity, *g*(*Y*) is the expected value of *Y *conditioned on the values of *X_j_*. For ER status, a categorical phenotype, *g*(*Y*) is the logit of the probability of being ER positive, given the values of *X_j_*.

The *β_j _*are coefficients that are related to the association between the probe set measurements and the phenotype, which we must estimate from the data. Intuitively, *β_j _*will be large in magnitude if the measurements in probe set *j *are strongly associated with the phenotype and will be zero if there is no such association.

The Lasso is a technique to estimate these *β_j _*given the data. Note that estimating *β_j _*goes hand in hand with feature selection; those genes for which we set the estimate of *β_j _*equal to zero are the ones we do not include in our set of selected features. In the Lasso, the estimates of the *β_j_*, ${\hat{\beta }}_{j}$, are those that maximize the log-likelihood subject to a penalty on the sum of the absolute values of the *β_j _*estimates (an *L*_1_-norm penalty); the geometry of this constraint causes the estimates of many of the *β_j _*to be set equal to exactly zero. We specify the severity of this penalty through a *shrinkage parameter λ*; the number of nonzero *β_j _*estimates is related to *λ*. We select *λ *through the cell line data; we describe how to select the value of *λ *in the Parameter Optimization subsection that appears later in this section.

**• SNSS**. This technique involves a similar setup as the Lasso: we adopt the same general linear model (1) and given the data, we estimate the *β_j _*coefficients. But unlike the Lasso, which performs feature selection through penalization (soft thresholding), SNSS does it through hard thresholding. SNSS is a forward stepwise procedure subject to a hard threshold on the number of genes; at each step, we add the gene with the strongest predictive signal to the set of selected features. We have the option of adding a single gene with a substantial predictive signal at each step, or a pair of genes: a gene with predictive signal and its most negatively correlated gene. Also, in SNSS, in order to avoid overfitting to the cell line data, we set *β_j _*equal to 1 or -1 according to the direction of association between the phenotype and the probe set measurement.

**• Regularized Discriminant Analysis (RDA) **[16]. Whereas in the Lasso and SNSS, we perform feature selection based on the general linear model (1), in RDA, we select our genes based on classifications according to the distributions of the sub-populations as defined through the various phenotypes.

RDA is a variant from Linear Discriminant Analysis [17] suitable for high-dimensional cases. We define sub-populations according to the different phenotypes; for instance, dacetuzumab sensitive samples comprise one sub-population, dacetuzumab semi-sensitive ones form another, and dacetuzumab resistant ones a third. For the purposes of this particular technique, we assume that for each sub-population, the distribution of the probe set measurements is approximately multivariate Normal; the means (centroids) vary across sub-populations, but the variances do not. Given the data, we estimate the distributions that characterize each sub-population.

In order to perform feature selection here, we look only for probe sets whose mean expression differ substantially between sub-populations by imposing a soft-thresholding step similar to the one used in the Lasso. Like in the Lasso, we control the degree of this soft-thresholding through a shrinkage parameter Δ. This procedure, however, also needs a shrinkage parameter for the sub-population distribution variances; often, in high-dimensional cases, the number of dimensions exceeds the number of samples, which would result in computational problems. To remedy this, we use a shrunken estimate of the variances; we control the degree of this shrinkage through the covariance matrix parameter *α*. We also need to find appropriate values of *α *and Δ given the data; we describe this in further detail in the Parameter Optimization subsection that appears later in this section.

### Model Fitting

After we use the Lasso, SNSS, or RDA for feature selection, we use a classification technique to derive a classification rule based on the selected genes. Here, we consider three options:

**• Use the model for feature selection for classification also**. Suppose we have the probe set measurements $X_{1}^{*},X_{2}^{*},\ldots X_{p}^{*}$ for a new sample. If we had used the Lasso and SNSS for feature selection, then given our estimates of *β_j_*, ${\hat{\beta }}_{j}$, we use the quantity

(2) $$\sum_{j=1}^{p}{\hat{\beta }}_{j}X_{j}^{*}$$

to predict the new sample's phenotype.

For ordered phenotypes such as dacetuzumab sensitivity, we assign the new sample to the phenotype whose numeric coding *Y *is closest to the quantity (2). In the context of the dacetuzumab sensitivity example, since *Y *= 0 denotes sensitivity, *Y *= 1 semi-sensitivity, and *Y *= 2 resistance, high values for (2) should correspond to dacetuzumab resistance, low values to sensitivity, and intermediate values to semi-sensitivity. Thus, we would classify this new sample as dacetuzumab sensitive if (2) is below a prescribed threshold, resistant if this quantity is above another higher threshold, and semi-sensitive otherwise.

For categorical phenotypes such as ER status, (2) becomes an estimate of the logit probability that the new sample is of the phenotype corresponding to *Y *= 1, namely an estimate of the logit probability that the new sample is ER positive. Therefore, if (2) is above a certain threshold, we classify the new sample as ER positive; otherwise, we classify the new sample as ER negative.

Meanwhile, for RDA, recall that in the feature selection step, we defined sub-populations according to the phenotypes and then estimated the distribution of the probe set measurements for each sub-population. Given the probe set measurements for the new sample, we determine the probability that $X_{1}^{*},X_{2}^{*},\ldots ,X_{p}^{*}$ came from each sub-population's multivariate normal distribution. The sub-population, and consequently the phenotype, we assign to the new sample is the one corresponding to the highest such probability.

**• Construct a K-Nearest Neighbors (KNN) **[5]** classifier based on only the selected genes**.

Here, we classify a new sample according to the phenotype of the cell line whose expressions of the selected genes are closest in Euclidean distance.

**• Construct a Random Forests **[6]** classifier based on only the relevant genes**. We construct an ensemble of *B *classification trees [5] using subsets of the genes we selected from the feature selection step. We apply each of these *B *trees to the probe measurements of a new sample, producing *B *classifications (one for each tree). The final classification is decided according to a majority vote among these *B *classifications.

### Parameter Optimization

Part of the classifier construction procedure involves the selection of appropriate *regularization parameter *values. All three feature selection techniques we consider; the Lasso, SNSS, and RDA; involve regularization parameters that we must select. Lasso requires a choice for the shrinkage parameter *λ*, SNSS requires a choice of the number of variables to include in the classifier, and RDA requires choices for two shrinkage parameters: *α *for the covariance matrices of the sub-population distributions, and Δ for the soft-thresholding.

The choice of parameter values is a key determinant of prediction performance. If models are *underfit *either by selecting too few genes or tightly constraining coefficient optimization, prediction performance can fall short of the best achievable. Alternatively, models that are *overfit*, which include too many genes without appropriate constraints on coefficient fitting will tend to interpret the data points in the training sample, essentially fitting to noise, so that prediction performance will be poor on validation samples. The Lasso is an example of a method in which one parameter constrains both the number of genes and the degree coefficient fitting. SNSS in contrast cannot overfit coefficients, which are set to -1 and 1, so only the number of genes can be constrained. Nevertheless, both models will interpolate the data when fitted with lax constraints.

We find appropriate values for the parameters based on the cell line data; specifically, we find the ones that result in the lowest generalization error. We generate a list of candidate parameter values and estimate the expected generalization error resulting from each one; finally, we select the one parameter value that results in the lowest estimated generalization error. We obtain estimates of the generalization errors through out-of-bag (OOB) [18] errors by applying the following procedure to the cell line data:

**for **each candidate parameter value **do**

**   for ***b *= 1, 2, ..., *B ***do**

      training samples ← cell lines sampled *N *times with replacement

      out-of-bag samples ← cell lines not included among training samples

      selected variables ← variables selected via application of feature selection technique (e. g. Lasso, SNSS) to training samples

      classifications ← predicted phenotypes of out-of-bag samples given selected variables

   end for

   final classifications ← final classification of each cell line by majority vote over *B *iterations of above procedure

   optimal parameter value ← parameter value corresponding to lowest out-of-bag error

end for

**Figure 3:**
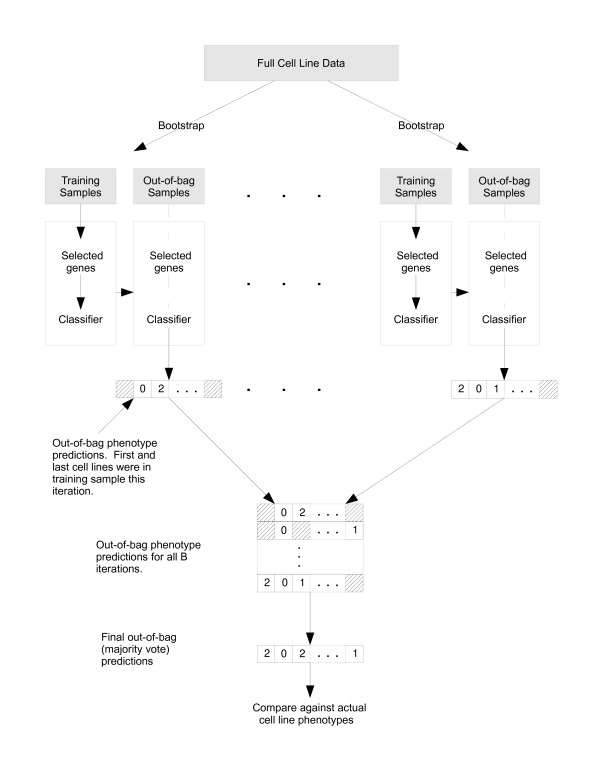
**Out-of-bag error computation schematic**. A schematic to illustrate the out-of-bag error computation procedure to determine the optimal regularization parameter value(s) for the Lasso, RDA, or SNSS, in the context of the dacetuzumab sensitivity classification case study. We repeat the following *B *times. We sample the *N *cell lines in the full cell line data *N *times, thus creating a bootstrap data set that will serve as our training sample. The approximately 37% of the cell lines left out of the bootstrap data set as a result of the sampling procedure will serve as an out-of-bag sample. We perform feature selection and construct a classifier using the bootstrap data, which we use to classify each of the cell lines in the out-of-bag sample as dacetuzumab sensitive (0), semi-sensitive (1), or resistant (2). For each cell line, we determine its final out-of-bag sensitivity classification by tallying up the classifications we obtained during the times the cell line appeared in the out-of-bag sample and selecting the sensitivity according to a majority vote. We compare the final out-of-bag classifications to the true sensitivities to determine the out-of-bag error.

We also illustrate this algorithm in Figure 3.

We then develop the final classifier by applying the Lasso, SNSS, or RDA, in conjunction with one of the model fitting techniques, to the full cell line dataset using the selected parameter value.

For the remainder of this paper, we notate classifiers as (feature selection technique used)/(classification technique used). For example, a classifier where we use the Lasso for feature selection and KNN for classification will be Lasso/KNN, whereas one where we use RDA for feature selection and the RDA model for classification is RDA/RDA.

## Results

### First Case Study: Dacetuzumab Sensitivity Classification

We apply each feature selection techniques in combination with a classifier to the cell line data to construct dacetuzumab sensitivity classifiers, which we then apply to independent patient data, classifying each patient as dacetuzumab sensitive, semi-sensitive, or resistant. We assess the performance of the classifiers against the patients' clinical outcomes.

Ideally, the predictions would be compared to patient responses to dacetuzumab. However, due to the pre-clinical initiation of the companion diagnostics development, no clinical data on dacetuzumab sensitivity were available for this analysis. As we have discussed in the Background section, the patients sensitive to dacetuzumab are expected to be predominantly of GCB subtype and have better survival rates, whereas the resistant patients are expected to be predominantly of ABC subtype and have poorer survival rates. Thus in this case study, the relative performance of classifiers may be assessed using prognostic information.

Recall that we consider three different feature selection techniques, the Lasso, SNSS, and RDA, in combination with three different classification approaches: (1) using the same model for classification as for feature selection, (2) using the selected features in a KNN classifier, or (3) using the selected features in a Random Forests classifier. Thus, we have constructed a total of nine classifiers. In order to assess the performance of each classifier, we compare prediction of sensitive, semi-sensitive, and resistant class membership to survival and to GCB/ABC/unknown subtypes.

Prior to our analysis, we perform some necessary pre-processing steps for the purposes of quality control. First, we filter out probe sets with high relative within-to-between biological replicate variability [19], which can be shown to be related to correlation between paired replicates. High variability within replicates on a biological sample matters only when that variability is large relative to the variability across biological samples, and if this ratio is too high, then the Pearson and Spearman correlation between replicate pairs will be low, indicating that the biological samples cannot be consistently ranked. Without consistent rankings from the predictors, good classification is not possible. We also filter out probe sets whose expression levels have both too little variability and too low intensity among the cell lines [20,21].

Although it is possible that some of the probes that are filtered out during this step could potentially measure activity of a biologically relevant process, the purpose here is to filter out probes that are not well-measured and keep probes that are. This step is expected to improve the performance of our techniques without introducing bias into our results. Bourgon et al [22] demonstrate that filtering based on statistics that are independent of the test statistic; as is the case here since the probe set intensity and variability levels are independent of the sample phenotypes; will result in an increase in power to detect differentially expressed genes while maintaining proper control of the rate of detection of spurious genes.

For each probe set, we compute a ratio of the variance of the expression levels between replicates to the expression variance between cell lines, and we keep only probe sets for which this ratio is sufficiently low; the threshold we use is the first quartile of this ratio among all probe sets. We then computed the median and mean absolute deviation of the expression levels of each probe set among the 31 cell lines; the probe sets we kept consisted of those whose mean expression levels were in the top quartile among all of the probe sets, or whose expressions had variances in the top quartile. These pre-processing steps reduced the number of candidate features for selection from 50 thousand to about 4 thousand.

Then we eliminate from the pool of probe sets available for selection for our classifiers all those not common to both cell line and patient data; recall that the cell line genomic data were measured on an Affymetrix U133Plus2.0 platform and the patient data were measured on U133A. In this step, we screened out another 1500 probe sets, leaving us with 2500. Finally, we center and scale both the cell line and patient data such that the mean and variance of the expressions of each probe set across samples in each data set are zero and one, respectively.

**Figure 4:**
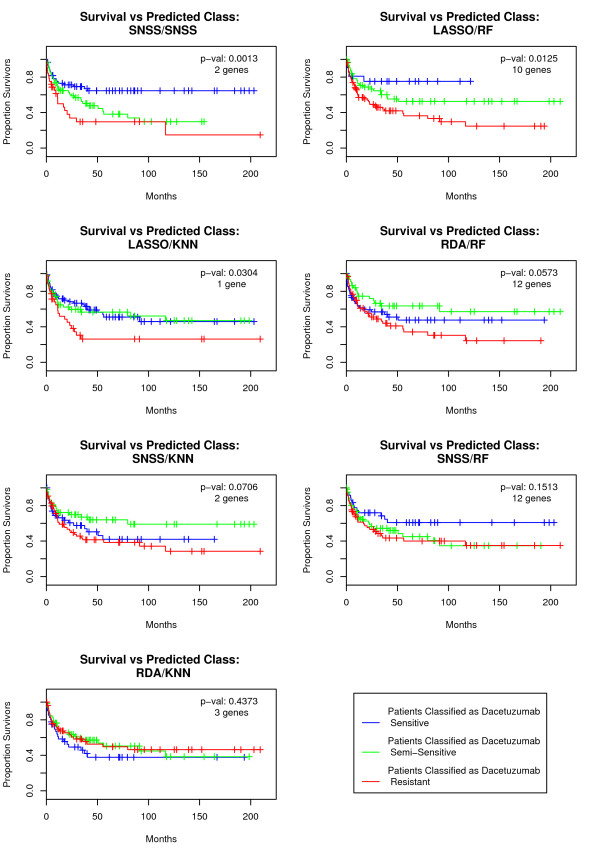
**Kaplan-Meier plots: survival rates of patients classified as dacetuzumab sensitive, semi-sensitive, and resistant by each of our classifiers**. Blue curves indicate dacetuzumab sensitive patients, green curves semi-sensitive, and red curves resistant. We omit two classifiers that involved no genes and hence classified all patients with the same dacetuzumab sensitivity.

Table 1 and Figure 4 summarize the survival rates of patients assigned to each dacetuzumab sensitivity class by each of the nine classifiers. Figure 4 depicts Kaplan-Meier plots for each sensitivity classification for seven of the classifiers. Note that we have excluded the Lasso/Lasso and RDA/RDA classifiers; no genes were selected for these two classifiers, and they classify all patients as dacetuzumab resistant. Table 1 lists the genes involved in each classifier alongside the p-values for log-rank tests for equality in survival rates between dacetuzumab sensitive and resistant patients and between patients in all three sensitivity classes.

**Table 1:** Differences in survival between patients assigned to each dacetuzumab sensitivity class according to each classifier.

Classifier	Selected Genes	Sens/Semi/Resis Log-rank p-values	Sens/Resis Log-rank p-values
SNSS/SNSS	2 genes total: RGS13, HSP90B1	0.0013	7 × 10^-4^

Lasso/RF	10 genes total: RGS13, CRTC3, PRPSAP2, PVRIG, SORD, WIPF1, CSNK2A2, GNB5, ERAP1, CAMSAP1	0.0125	0.0212

Lasso/KNN	1 gene total: RGS13	0.0304	0.0077

RDA/RF	11 genes total: GRK5, GNB5, IFITM1 (2 probe sets selected), CSNK2A2, SCARB1, UGDH, MSH2, GORASP1, PECI, VPS54, WWOX	0.0573	0.34

SNSS/KNN	2 genes total: RGS13, HSP90B1	0.0706	0.4661

SNSS/RF	12 genes total: RGS13, HSP90B1, PRPSAP2, IFITM1, SORD, PYROXD1, EVI2B, ZNF322A, NAGK, BTG2, RAB13, DPYD	0.1513	0.0556

RDA/KNN	2 genes total: GRK5, IFITM1 (2 probe sets selected)	0.4373	0.3035

Lasso/Lasso	No genes selected	-	-

RDA/RDA	No genes selected	-	-

The genes selected by each classifier are summarized alongside p-values for the log-rank tests for differences between the survival rates of dacetuzumab sensitive, semi-sensitive, and resistant patients, and for the two-way difference between the survival rates of dacetuzumab sensitive and resistant patients only.

A key difference between the classifiers involves the prognoses of the patients assigned to the semi-sensitive class. For example, for the SNSS/SNSS classifier, the survival rates of these patients more closely resemble those of the resistant classification, whereas for the Lasso/RF classifier, these survival rates are more similar to those of the sensitive classification.

Table 2 shows the proportions of GCB, ABC, and unknown DLBCL subtype malignancies among patients in each dacetuzumab sensitivity class as assigned by the seven classifiers where at least one gene was selected. A very high proportion of predicted dacetuzumab sensitive patients are of the GCB subtype; this proportion is higher than 80% for the most successful of the classifiers. Also, for the most successful classifiers, a large proportion of predicted dacetuzumab resistant patients are of the ABC subtype. For example, 54% of those patients that the SNSS/SNSS classifier predicted to be dacetuzumab resistant are of the ABC subtype, whereas only 20.8% of these patients are of the GCB subtype. For the Lasso/RF classifier, 47% of the patients classified as dacetuzumab resistant are of the ABC subtype, and 36% of these patients are of the GCB subtype. Finally, for most of these classifier, the patients classified as dacetuzumab semi-sensitive were largely of the GCB subtype; one notable exception to this is the SNSS/SNSS classifier, where the subtypes are GCB and ABC subtype proportions are more even.

**Table 2:** Predicted dacetuzumab sensitivity versus GCB/ABC subtype

Classifier	Odds Ratio/95% CI		GCB	ABC	Unknown
Lasso/RF	∞	Sens	0.824	0	0.176
		
		Semi	0.655	0.167	0.179
		
		Resis	0.359	0.427	0.214

SNSS/SNSS	208 (25.40, 1703.23)	Sens	0.889	0.011	0.100
		
		Semi	0.361	0.373	0.265
		
		Resis	0.208	0.542	0.250

SNSS/RF	81.71 (10.50, 635.96)	Sens	0.898	0.020	0.082
		
		Semi	0.625	0.205	0.170
		
		Resis	0.250	0.464	0.286

SNSS/KNN	29.03 (6.53, 129.17)	Sens	0.769	0.031	0.200
		
		Semi	0.591	0.303	0.106
		
		Resis	0.344	0.400	0.256

Lasso/KNN	6.38 (2.69, 15.16)	Sens	0.613	0.132	0.255
		
		Semi	0.600	0.338	0.062
		
		Resis	0.320	0.440	0.240

RDA/RF	4.31 (1.91, 9.72)	Sens	0.657	0.157	0.186
		
		Semi	0.612	0.194	0.194
		
		Resis	0.393	0.405	0.202

RDA/KNN	2.31 (0.95, 5.61)	Sens	0.661	0.161	0.179
		
		Semi	0.477	0.295	0.227
		
		Resis	0.532	0.299	0.169

The odds ratio for GCB subtype by dacetuzumab sensitive versus resistant classification, with a 95% confidence interval. The GCB/ABC/Unknown subtype distribution is shown also for the semi-sensitive class, this class is excluded from the odds ratio calculation for simplicity. Two of the classifiers, Lasso/Lasso and RDA/RDA, selected no genes and have therefore been excluded from this table.

Table 2 also provides odds ratios, with 95% confidence intervals, comparing the odds that a patient classified as sensitive has a GCB subtype malignancy to the odds that patient classified as resistant has a GCB subtype malignancy. For simplicity, the semi-sensitive class is excluded from this summary statistic. Out of all seven classifiers listed in Table 2, only one (RDA/KNN) had a confidence interval that contained one; for the other six, we have strong evidence for an association between classification and subtype.

Some of the most commonly recurring genes in these classifiers include RGS13, HSP90B1, and IFITM1. Regulator of G-protein-mediated signal transduction 13 (RGS13) is a nuclear factor that suppresses CREB-dependent gene expression [23] expressed predominantly in germinal center B-cells and thymic epithelial cells [24]. These findings would be consistent with the our observations that RGS13 is expressed at higher levels in the GCB subtype of NHL cell lines. Furthermore, Heat shock protein 90 kDa beta, member 1 (HSP90B1) is a member of the HSP90 family of proteins. Intriguingly, HSP90B1 has been found to play a role in the regulation of MHCII antigen presentation in B cells of exogenous and endogenous antigens [25] and could explain the increased expression observed in the ABC subtype of NHL cell lines. Interferon induced transmembrane protein 1 (IFITM1) is induced by IFN-gamma and functions to prevent the proliferation of cells infected with virus via stabilization of the tumor suppressor, p53 [26]. Higher expression levels of IFITM1 were also found to be associated with improved survival of CML patients [27]. The increased expression in the ABC subtype cell lines we observed makes biological sense since hyperproliferation and somatic mutation are events that are no longer required for a B-cell that will be selected for maturation.

Other commonly recurring genes include G protein-coupled receptor kinase 5 (GRK5), which is stabilized by HSP90 [28] and plays a role in activation of the NFkappaB signaling pathway by phosphorylating key residues of IKKbeta [29]; CREB regulated transcription coactivator 3 (CRTC3), a transcriptional coactivator for CREB as part of the TORC signaling pathway [30]; and Phosphoribosyl pyrophosphate synthetase-associated protein 2 (PRPSAP2), a non-catalytic subunit of the phosphoribosylpyrophosphate synthetase enzyme, which is an essential part of purine, pyrimidine, nucleotide, histidine, tryptophan and NAD synthesis [31].

Two genes, LMO2 and CD44, were not selected in any of these models but are components of previously published GCB/ABC classifiers [32]. Burington et al (Burington, B., Yue, P., Shi, X., et al: **CD40 Pathway Activation Status Predicts the Anti-Tumor Activity of CD40 Therapy in Diffuse Large B-Cell Lymphoma**, submitted) hypothesize that dacetuzumab sensitivity in tumor B-cells is associated with an inactive CD40 pathway, which in turn is characteristic of normal germinal center B-cells, while the CD40 pathway in normal activated B-cells is active. While there is a strong association between the two classifiers and a biological relationship to an important pathway in B-cell maturation, the classifier trained using dacetuzumab sensitivity may be more focused on the CD40 pathway and is a better predictor of sensitivity.

### Second Case Study: ER Status Classification

The same methodology is used to construct ER status classifiers from cell line data. The published patient data also contain ER status information [10].

We performed pre-processing steps in a manner similar to the dacetuzumab sensitivity case study. Unlike in the first case study, here, technical replicates of the cell line data sets were not available, so screening by relative within-to-between replicate variability was not possible; here, we only screened out all probe sets whose expressions among the cell lines had both low variability and low intensity level. We computed the median and mean absolute deviation of the expression levels of each probe set among the 47 cell lines; the probe sets we kept consisted of those whose median expression levels were in the top quintile among all of the probe sets, or whose expressions had mean absolute deviations in the top quintile. These pre-processing steps reduced the number of candidate features for selection from 22 thousand to about 7 thousand. Here, screening out probe sets not common to the cell line and patient data was not necessary; both the patient data and cell line data were measured on U133A platforms. Finally, we centered and scaled the probe set expressions in both the cell line and patient data. A similar approach was used in Chin et al [10].

Again, we have constructed nine ER status classifiers using the different combinations of feature selection and classification techniques, and we assess each of them by comparing the classifications with known phenotypes in the independent patient data. Table 3 summarizes the performance of the ER status classifiers. The misclassification rate here is defined as the proportion of patients whose classifications do not match their known phenotypes. For the most successful classifiers, this misclassification rate is approximately 10%. The classifiers with the lowest misclassification rates involve only one or two genes, and these classifiers all involve ESR1, which encodes estrogen receptor.

**Table 3:** Performances of ER status classifiers

Classifier	Selected Genes	Patient Prediction Error
SNSS/SNSS	1 gene total: ESR1	0.1017

Lasso/KNN	1 gene total: ESR1	0.1186

Lasso/RF	1 gene total: ESR1	0.1186

SNSS/RF	2 genes total: ESR1, TOM1L1	0.1186

SNSS/KNN	2 genes total: ESR1, TOM1L1	0.161

Lasso/Lasso	19 genes total: ESR1, TOM1L1, CPT1A, SRPR, APOD, COIL, CYB561, C10orf116, ST6GALNAC2, MICALL1, ABAT, FBP1, CA12, JAG1, PDCD4, FXYD5, RSAD1, C14orf132, MRPL35	0.1864

RDA/RDA	7 genes total: ESR1, EIF3D, CPT1A, COIL, INPP4B, SIAH2, RSAD1	0.4661

RDA/RF	No genes selected	0.5254

RDA/KNN	1 gene total: CPT1A	0.5254

Summaries of the performances of the nine ER status classifiers, including the selected genes and the patient ER status prediction error.

## Discussion

In each of the case studies, we were able to construct at least a few low-dimensional classifiers that generalized well from cell line data to patient data using some combination of feature selection and classification techniques.

The patient ER status assignments from some of the classifiers, particularly SNSS/SNSS and Lasso/RF, had low misclassification rates. For the most successful ER status classifiers, this misclassification rate was about 10% to 12% and only one or two genes were required. Meanwhile, some of the dacetuzumab sensitivity classifiers, notably SNSS/SNSS and Lasso/RF, had good prognostic ability. For these classifiers, dacetuzumab sensitivity classifications seemed to overlap with the prognostic GCB versus ABC DLBCL subtype; patients classified as dacetuzumab sensitive were largely of the GCB subtype whereas those classified as dacetuzumab resistant were largely of the ABC subtype, and this is evident in both the difference in survival rates between the dacetuzumab sensitive and resistant classes (Figure 4 and Table 1) as well as the strong association between GCB versus ABC subtype and dacetuzumab sensitivity and resistance (Table 2). Because of the coincidence of the prognostic and predictive pathways in this particular case, these classifiers are hypothesized to have good predictive ability as well. This hypothesis is currently being tested in a randomized Phase II trial in second line DLBCL.

Note that we assess the performance of our classifiers primarily in terms of the sensitive and resistant classes. Even for the most successful classifiers in this case study, prognoses for patients classified as dacetuzumab semi-sensitive still remained somewhat mixed. Semi-sensitive cell lines were somewhere in between sensitivity and resistance, sharing expression patterns with both types, and this makes them difficult to identify correctly. During the out-of-bag error computations steps in the construction of each classifier, we rarely classified dacetuzumab sensitive cell lines as resistant or vice versa; two notable outlying exceptions to this were the cell lines SU.DHL.8 and Karpase.422. We did observe some sensitive and resistant cell lines being misclassified as semi-sensitive and semi-sensitive cell lines frequently being classified as sensitive or resistant. Most of the out-of-bag classification errors were a result of these semi-sensitive cell lines.

This is also reflected in the patient data. The survival rates of patients classified as dacetuzumab semi-sensitive sometimes resembled the survival rates of those classified as sensitive, as what happened with the Lasso/KNN classifier. In other cases, such as the SNSS/SNSS classifier, the survival rates of patients classified as semi-sensitive more closely resembled those of the resistant class. The distribution of GCB versus ABC subtypes among patients classified as semi-sensitive also is somewhat ambiguous; for some classifiers, such as Lasso/RF, Lasso/KNN, and SNSS/RF, the semi-sensitive patients are predominantly of the GCB subtype, whereas for others, such as the SNSS/SNSS classifier, a much larger proportion of the semi-sensitive patients are of the ABC subtype.

Because of the ambiguous nature of semi-sensitive samples, combining them with either resistant or sensitive samples is not likely to be helpful. Nevertheless, in terms of constructing the classifiers themselves, they were helpful as a separate ordered category since many outcomes trended on average from sensitive through semi-sensitive to resistant. During gene selection, some genes exhibited trending intensity through the semi-sensitive cell lines and excluding these cell lines did not improve the performance of gene selection or classification in this application (excluding intermediate samples is a technique worth trying). Also, the semi-sensitive class may provide insight into the relative positive versus negative predictive value of competing classifiers and therefore whether they would be more useful for maximizing the response rate or excluding non-responders in clinical practice. Classifiers such as SNSS/SNSS, where the prognoses of patients classified as semi-sensitive more closely resemble those of patients classified as resistant, have high positive predictive value and are best for maximizing the response rate. Meanwhile, classifiers such as Lasso/RF and SNSS/RF, where the prognoses of patients classified as semi-sensitive more closely resembles those of patients classified as sensitive, have high negative predictive value may be best suited to settings that require high confidence in predictions of non-response.

Here, we introduce SNSS, a novel forward stepwise procedure that is designed to model either up/down regulated pathways or pathways better represented by independent probe selection. The two case studies illustrate each approach. The former approach is more appropriate when unsupervised clustering suggests the presence of multi-gene up-to-down regulated contrasts, for example appearing as a red versus green four-square in a heat map. It modifies the stepwise procedure to add anti-correlated pairs rather than choosing the single strongest predictor. The latter approach is essentially forward stepwise, which is more appropriate for situations where one or more pathways are distinctly characterized by dysregulation of one or a few genes (e.g. FGFR3 and MMSET in t(4;14) multiple myeloma [33]). Future work will extend the model to include both types of pathways in a single model and generalize the forward stepwise approach to the addition of small clusters of positively and negatively correlated genes.

In these case studies, SNSS performed well in both feature selection and model fitting. In particular, SNSS with SNSS, as well as Lasso with Random Forests, produced classifiers that were successful in both generalizing from the cell line to the patient data and requiring only a small number of genes. These results, however, are not sufficient to suggest that these two combinations are inherently the best feature selection and classification techniques for deriving classifiers from cell line data. This simply suggests that these two combinations were the best for these particular case studies. The characteristics of these data sets may have just been conducive to the success of these two combinations. In data involving more cell lines, for example, the SNSS/SNSS and Lasso/RF classifiers may be outperformed by others.

Answering the question of when a particular classifier will or will not perform well requires further research. SNSS may perform better as a feature selection technique when the size of the training data is small, the data contain a large number of correlated variables, and the platform on which the patient assay is implemented differs from the cell line platform used for assay development (i. e. non-identical distributions of the gene expression measurements). Under these circumstances, the hard-thresholding of SNSS based on simple signed averages may match or outperform more complex fitting procedures such as Lasso. On the other hand, if the patient and cell line genomic data are measured on identical platforms and the size of the training set were larger, more complex fitting techniques may improve the classification accuracy; in these conditions, SNSS-based classifiers may not perform as well.

However, RDA may not be the best feature selection method for the purposes of constructing PCR-based assays. Both the Lasso and SNSS are inherently effective at keeping the number of genes involved small. The Lasso can only select at most *N *genes, where again, *N *is the number of cell lines, and since in these types of situations, *N *is usually small, the number of genes involved in a Lasso-based classifier is also generally small. In SNSS, we directly control the number of genes involved. Guo et al provide simulation studies that show that RDA can, and often does, select more than *N *genes [16].

Although we use linear models for feature selection and classification, none of our reported estimates of accuracy depend upon whether or not a linear model holds strictly. Approximate lognormality is well supported by descriptive analyses of these data (not shown for reasons of space) as well as being common in the literature. It is well known that the signal properties of microarrays are skewed and that using the log signal improves the signal properties by reducing heteroskedacity [34]. The linear methods that we used for the most part performed reasonably well with roughly approximately normal data. It is important, however, that our assessment of their performance does not rely on the model assumptions, because violations can impact model performance. For example, for RDA spherical distributions of the data within class and for the Lasso normality of the errors improve the modelfit. In addition, marked violations of assumptions required for valid inference, such as homoskedasticity, may lessen the optimality of feature selection procedures that use linear models. We believe that these are all good reasons to compare approaches in applied settings, as we do in this manuscript.

## Conclusions

Our primary aim was to develop predictive or prognostic assays from cell line genomic data that not only generalize well to patient data but are also feasible to implement in practice; in other words, we wanted to develop assays that not only assign phenotypes to patients given their genomic data with high accuracy, but also involve a small number of genes. To this end, we applied feature selection techniques in conjunction with classification methods to cell line data to (1) find a small subset of the most important genes, and then (2) develop an appropriate classification rule given the expression levels of these genes. We assessed these classifiers by applying them to independent patient genomic data.

For each of our case studies, we constructed nine different classifiers using different combinations of feature selection and classification techniques. Not all of the feature selection and classification method combinations we considered produced classifiers that both generalized to patient data well and involved a small number of genes, but in each case study, we were able to produce at least a few classifiers that were successful in this regard. In particular, SNSS with SNSS and Lasso with Random Forests produced low-dimensional classifiers that assigned patient phenotypes with high accuracy in each of the cases studied here.

These results demonstrate the feasibility of combining feature selection techniques such as the Lasso, SNSS, and RDA with classification methods such as KNN and Random Forests in order to develop assay based on genomic measurements from cell lines that subsequently exhibit good performance in patients. Even though two particular feature selection and classification method combinations performed well in these case studies, in other settings, different combinations may outperform them. The question of what general conditions determine the relative performance of the combinations remains open.

## Appendix

### The Lasso

Denote *Y_i _*as the value of *Y *and *X_ij _*the value of *X_j _*for sample *i*. The Lasso estimates of the *β_j _*are the *β_j _*that minimize

(3) $$\sum_{i=1}^{N}{\left[Y_{i}-\sum_{j=1}^{p}X_{ij}\beta_{j}\right]}^{2}+\lambda \sum_{j=1}^{p}\left|\beta_{j}\right|$$

for some shrinkage parameter *λ *≥ 0. The first term of (3) encourages ${\hat{\beta }}_{j}$ values that provide a good fit to the data and the second term performs feature selection and regularizes the minimization problem.

Without the second term, the minimization problem is ordinary least squares [5], which is degenerate when *p *≫ *N *as is the case in these two case studies, since the data can then be interpolated. Also, the solution to the ordinary least squares minimization problem has ${\hat{\beta }}_{j}$ = 0 with zero probability, so this minimization does not perform feature selection.

The geometry of the *L*_1_-norm penalty, the second term in (3) sets ${\hat{\beta }}_{j}$ equal to exactly zero for many *j*, thus performing feature selection simultaneously with fitting. The number of nonzero ${\hat{\beta }}_{j}$ is controlled directly through *λ*; small values of *λ *result in low weight to the *L*_1_-norm penalty and therefore many nonzero ${\hat{\beta }}_{j}$ estimates, or many variables being selected, and as *λ *increases, so does the weight of the penalty. The number of selected variables consequently decreases.

The Lasso will select at most *N *variables [4].

### RDA

RDA is a variant of Linear Discriminant Analysis (LDA) [17] which is particularly useful for cases where *N *≫ *p*. Again, we define sub-populations based on the phenotypes; for each sub-population, the gene expressions have their own multivariate Normal distribution. We assume the variances Σ do not differ between sub-populations, but the mean gene expressions *μ_k _*do. LDA is unstable for when *N *≫ *p *since $\hat{\Sigma }$, the maximum likelihood estimate of the variance will not be full rank in this case; we resolve this problem in RDA by using

(4) $$\tilde{\Sigma }=\;\alpha \hat{\Sigma }+(1-\alpha )I_{p}$$

in place of $\hat{\Sigma }$, where 0 ≤ *α *< 1 and *I_p _*is the *p *× *p *identity matrix.

Additionally, in RDA we may shrink each element of ${\tilde{\Sigma }}^{-1/2}{\hat{\mu }}_{i}$ by some Δ > 0, which will perform gene selection [16]. More specifically, let *z_i _*be the *i^th ^*element of ${\tilde{\Sigma }}^{-1/2}{\hat{\mu }}_{i}$; then we replace ${\tilde{\Sigma }}^{-1/2}{\hat{\mu }}_{i}$ with a vector whose *i^th ^*element is given by

(5) $$z_{i}^{*}=\text{sgn}\left(z_{i}\right){\left(\left|z_{i}\right|-\Delta \right)}_{+}$$

### SNSS

As mentioned before, at each step of SNSS, we have the option of adding to the predictive model a single gene with predictive signal or a pair of genes: a gene with predictive signal and its most negatively correlated gene. In either case, the main gene is selected according to the strength of its correlation with the residuals; if we choose to select pairs of genes at each step, the other is the gene whose expression is most negatively correlated with that of the main gene. Also, unlike Lasso and RDA, the values of ${\hat{\beta }}_{j}$ we obtain from SNSS are restricted to {-1, 0, 1}.

Define *m_k _*as the index of the main gene of the *k^th ^*pair and *p_k _*as the index of the corresponding pair gene. Define the residual of sample *i*, *R_i_*, as

(6) $$R_{i}=Y_{i}-\sum_{k}\left[{\hat{\beta }}_{m_{k}}X_{im_{k}}+{\hat{\beta }}_{p_{k}}X_{ip_{k}}\right]$$

If no gene pairs have been selected, *R_i _*= *Y_i_*. Also, if we only opt to select single genes at each step, *β_pk _*always equals zero.

For the SNSS procedure, we need to specify *K*, the total number of steps to take. If we opt to select pairs of genes at each step, *K *is the number of gene pairs to include in the predictive model; otherwise, *K *is the number of genes to include. Then

*R_i _*← *Y_i _*for each *i *= 1, 2, ..., *N*

**for ***k *= 1, 2, ..., *K ***do**

   main gene ← gene whose expression has strongest correlation with *R_i_*, i. e., find *m_k _*= argmax*_j _*|Corr [*R_i_*, *X_j _*]| ${\hat{\beta }}_{m_{k}}$ ← sgn(Corr[*R_i_*, *X_j_*])

   **if **we are selecting pairs of genes **then**

      pair gene ← gene whose expression is most negatively correlated with main gene, i. e., find

      $p_{k}=\arg\min_{j}\text{Corr[}X_{m_{k}},X_{j}\text{]}$

      ${\hat{\beta }}_{p_{k}}\leftarrow -{\hat{\beta }}_{m_{k}}$

   end if

**   if **we are selecting pairs of genes **then**

      $R_{i}\leftarrow R_{i}-\left[{\hat{\beta }}_{m_{k}}X_{im_{k}}+{\hat{\beta }}_{p_{k}}X_{ip_{k}}\right]$

   else

      $R_{i}\leftarrow R_{i}-{\hat{\beta }}_{m_{k}}X_{im_{k}}$

   end if

end for

### KNN

Suppose that through the Lasso, RDA, or SNSS, we have selected the genes *X*_1_, *X*_2_, ..., *X_d_*. Here, we first identify the single cell line whose expressions for these *d *genes are closest to the patient's and then assign the patient's class according to the known phenotype of this cell line. In general, the patient classification can be determined using a majority vote among the *k *cell lines whose gene expressions are closest, but because of the small number of cell lines, we only use the single nearest cell line here. More specifically, for each cell line, we compute

(7) $$D((X_{1},X_{2},\ldots ,X_{d}),(X_{1}^{*},X_{2}^{*},\ldots ,X_{d}^{*}))$$

where ($X_{1}^{*},X_{2}^{*},\ldots ,X_{p}^{*}$) are the patient's expression of these *d *genes and *D *is a distance metric; in our case, we use Euclidean distance

(8) $$D((X_{1},X_{2},\ldots ,X_{d}),(X_{1}^{*},X_{2}^{*},\ldots ,X_{d}^{*}))=\sqrt{{\left(X_{1}^{*}-X_{1}\right)}^{2}+{\left(X_{2}^{*}-X_{2}\right)}^{2}+\ldots +{\left(X_{d}^{*}-X_{d}\right)}^{2}}$$

We then rank-order the distances and classify the patient according to the phenotype of the nearest-ranked cell line.

### Random Forests

Random Forests [6] is an ensemble classifier that consists of many decision trees and outputs the class which is the mode of the class outputs by the individual trees. Random Forests uses a random subset of features selected at each tree node, thus introducing additional sources of perturbation into the data compared to other re-sampling techniques such as bagging [18].

We repeat the following *B *times. We sample from the *N *cell lines *N *times with replacement to create a bootstrap data set. We then randomly select *m *out of the *d *genes selected through the Lasso, SNSS, or RDA and construct a classification tree using the expressions of these *m *genes based on the bootstrap data. Repeating this process *B *times will produce an ensemble of *B *trees *T*_1_, ..., *T_b_*.

Given a patient's expressions of the *d *selected genes, $X_{1}^{*},X_{2}^{*},\ldots ,X_{p}^{*}$, we obtain *B *classifications through the ensemble of trees, one for each tree:

(9) $$\begin{array}{l} C_{1}=T_{1}(X_{1}^{*},X_{2}^{*},\ldots ,X_{d}^{*})\\ C_{2}=T_{2}(X_{1}^{*},X_{2}^{*},\ldots ,X_{d}^{*})\\ \;\;\;\;\vdots \\ C_{B}=T_{B}(X_{1}^{*},X_{2}^{*},\ldots ,X_{d}^{*}) \end{array}$$

The final classification of this patient is determined by a majority vote among *C*_1_, *C*_2_, ..., *C_B_*.

## Competing interests

The authors declare that they have no competing interests.

## Authors' contributions

EH carried out the data analysis and wrote the manuscript. BB developed the SNSS procedure. JF, NLK, and BB assisted with the data analysis. DD designed and managed the dacetuzumab pre-clinical studies and provided the cell line data for dacetuzumab case study. XS performed the cell line experiments. JF provided the scientific background for the breast cancer case study. PY, DD, and BB provided the scientific background for the dacetuzumab case study. JF, NLK, PY, DD, and BB all reviewed and revised the manuscript. All authors have read and approved the final manuscript.

## Pre-publication history

The pre-publication history for this paper can be accessed here:

http://www.biomedcentral.com/1471-2407/10/586/prepub
